# Sex and fetal genome influence gene expression in pig endometrium at the end of gestation

**DOI:** 10.1186/s12864-024-10144-1

**Published:** 2024-03-21

**Authors:** Agnes Bonnet, Lisa Bluy, Laure Gress, Laurianne Canario, Laure Ravon, Aurelie Sécula, Yvon Billon, Laurence Liaubet

**Affiliations:** 1 GenPhySE, Université de Toulouse, INRAE, INPT, ENVT, 31326 Castanet Tolosan, France; 2 GenESI, INRAE, Le Magneraud, 17700 Surgères, France; 3 Present Address: IHAP, Université de Toulouse, INRAE, ENVT, Toulouse, France

**Keywords:** Perinatal survival, Feto-maternal interface, Endometrium, Fetal genome, Fetal sex, Piglets

## Abstract

**Background:**

A fine balance of feto-maternal resource allocation is required to support pregnancy, which depends on interactions between maternal and fetal genetic potential, maternal nutrition and environment, endometrial and placental functions. In particular, some imprinted genes have a role in regulating maternal-fetal nutrient exchange, but few have been documented in the endometrium. The aim of this study is to describe the expression of 42 genes, with parental expression, in the endometrium comparing two extreme breeds: Large White (LW); Meishan (MS) with contrasting neonatal mortality and maturity at two days of gestation (D90-D110). We investigated their potential contribution to fetal maturation exploring genes-fetal phenotypes relationships. Last, we hypothesized that the fetal genome and sex influence their endometrial expression. For this purpose, pure and reciprocally crossbred fetuses were produced using LW and MS breeds. Thus, in the same uterus, endometrial samples were associated with its purebred or crossbred fetuses.

**Results:**

Among the 22 differentially expressed genes (DEGs), 14 DEGs were differentially regulated between the two days of gestation. More gestational changes were described in LW (11 DEGs) than in MS (2 DEGs). Nine DEGs were differentially regulated between the two extreme breeds, highlighting differences in the regulation of endometrial angiogenesis, nutrient transport and energy metabolism. We identified DEGs that showed high correlations with indicators of fetal maturation, such as ponderal index at D90 and fetal blood fructose level and placental weight at D110.

We pointed out for the first time the influence of fetal sex and genome on endometrial expression at D90, highlighting *AMPD3*, *CITED1* and *H19* genes. We demonstrated that fetal sex affects the expression of five imprinted genes in LW endometrium. Fetal genome influenced the expression of four genes in LW endometrium but not in MS endometrium. Interestingly, both fetal sex and fetal genome interact to influence endometrial gene expression.

**Conclusions:**

These data provide evidence for some sexual dimorphism in the pregnant endometrium and for the contribution of the fetal genome to feto-maternal interactions at the end of gestation. They suggest that the paternal genome may contribute significantly to piglet survival, especially in crossbreeding production systems.

**Supplementary Information:**

The online version contains supplementary material available at 10.1186/s12864-024-10144-1.

## Background

Reproductive success depends on the fine balance between the nutrient demand for an optimal fetal growth and the maternal nutrient requirements to support the pregnancy. This balance takes place at the feto-maternal interface, defined as the interaction between the mother (uterus/endometrium) and the fetus (placenta). The endometrium is essential for female fertility, including implantation, placentation, embryonic development and maintenance of pregnancy. Many critical morphologic and functional changes take place in the endometrium throughout gestation such as the differentiation process of stromal fibroblasts into a decidual phenotype and the increase in uterine glandular secretions during embryo implantation [1, 2]. Numerous studies have focused on elucidating the expression and function of uterine genes during pregnancy but they have mainly referred to the implantation period [2, 3].

During the last decades, improving prolificacy rate and carcass merit (i.e. leaner meat) have been the main objectives of selection. As a result, the increase in litter size led to uterine crowding and increased nutrient requirements during gestation. Within-litter crowding in the uterine space is greatest in late gestation and can lead to competition for feto-maternal resources, resulting in differential growth between littermates [4]. Under these conditions, more piglets are likely to suffer from IUGR (intrauterine growth restriction) [5].

As a consequence of this genetic selection, perinatal mortality and within-litter variation in birth weight have increased, with consequences for postnatal growth [6]. In commercial pig herds, mortality from birth to weaning ranges from 10 to 20% [7]. This is not compatible with efficient and sustainable production. Last, several studies have showed that piglet maturity, defined as the complete development allowing survival at birth, is likely to be an important determinant of subsequent survival [8–11]. Accordingly, piglet maturation and maximum fetal and placental growth, which occur towards the end of gestation, result in a nutritional burden of pregnancy for the mother.

Despite the importance of the foeto-maternal interface in the fetal development, studies of the porcine endometrium have focused mainly on early gestation because of the high embryonic mortality. However, some interesting recent studies have provided insight into the uteroplacental function of the pig from mid to late gestation. Kim et *al.* [12] investigated the global gene expression profiles in the endometrium at different days of gestation (D). The study described some stage-specific gene expression patterns and 34 hub genes such as SPP1 (Secreted Phosphoprotein 1), an extracellular matrix protein (ECM) that binds receptors such as integrins to effect cell-cell and cell-ECM adhesion at the maternal-conceptus interface, participates in embryo/feto–maternal attachment and promotes signaling between these tissues [13]. At midgestation, Wang et *al.* [14] highlighted differential protein expression between Meishan (MS, rustic breed) and Duroc (commercial breed). Some of these proteins are involved in metabolic pathways such as arginine metabolism. Other proteins were components of the extracellular matrix or associated with extracellular vesicles such as exosomes that participated to the feto-maternal crosstalk. A change in the endometrial proteome at different gestational stages and between normal and intrauterine growth restricted (IUGR) pig fetuses was also reported by Chen et *al.* [15]. They underlined an increase in oxidative stress and apoptosis in the uteroplacental tissue and a decrease in proteins involved in energy metabolism, transport and vascularization in endometrium of the IUGR fetuses group compared to the normal fetuses group.

The intrauterine growth is regulated by genetic, epigenetic and environmental factors. Recently, Sferruzzi-Perri et *al*. [16] used genetic inactivation of the growth and metabolism regulator, Pik3ca (encoding PIK3CA, also known as p110α, α/+), to create reciprocal crosses in mouse species and provided evidence that maternal and fetal genomes interplay. They identified significant interactions between maternal and fetal genomes in determining placental capillary length and diameter at D16 (gestation period of 19-21 days). Maternal genotype also had a strong effect on placental expression of transporter and cell lineage genes. In cattle, Mansouri-Attia et *al*. [17] showed that the conceptus derived from in vitro fertilization (IVF) or somatic cell nuclear transfer (SCNT) at D20 (gestation length ranges from 279 to 287 days) modifies endometrial gene expression compared to an in vivo fertilized embryo. Petry et *al.* [18, 19] cited other examples of variation in the fetal genome created in mice and domestic animals or derived from human cohort studies that may affect maternal physiology such as maternal blood pressure in mice, maternal serum estrogen and progestagen concentrations in horses or maternal circulating triglyceride concentrations in human pregnancy. In pig species, piglet survival is considered as a maternally influenced trait for genetic selection and the contribution of the piglet genome appears to be underestimated.

In addition to the role of the fetal genome, recent literature in pig species has highlighted associations between fetal size, sex and endometrial integrin expression or cellular processes such as endometrial apoptosis, proliferation and angiogenesis, that are dependent on gestational age [20, 21].

Finally, at the feto-maternal interface, imprinted genes (monoallelic expression of genes based on their parental origin) have the role of driving communication and regulating nutrient exchange between the mother and the fetus. The maternally expressed genes are involved in mechanisms that limit resource transfer to the embryo while paternally expressed genes are involved in mechanisms that enhance such transfer [22]. Genomic imprinting has been mainly described in the placenta but few studies have investigated its potential role in the endometrium. O’Doherty et *al*. [23] observed an effect of the day of gestation (from Day 5 to 16) on the expression of several imprinted genes (*MEST* (Mesoderm Specific Transcript), *PEG3* (Paternally expressed 3), *PLAGL1* (Pleiomorphic adenoma gene-like 1)), and a tissue-dependent gene expression (Caruncular versus intercaruncular endometrial regions) for *MEST*, *H19* (H19 Imprinted Maternally Expressed Transcript), *PEG3* and *SNRPN* (Small Nuclear Ribonucleoprotein Polypeptide N) genes at D20 during the preimplantation period in cows (D5 to D20).

Considering this, the present study focused on 42 genes already identified in placenta, endometrium or pig muscle and with a genomic sequence in pigs (Additional file 1). This gene set includes 27 imprinted genes selected from the 489 imprinted genes present in mammalian species (https://www.geneimprint.com/site/genes-by-name, June 2023). Twelve genes were selected because their expression was influenced by one of the parental genomes in our previous study using the same experimental design [10]. Finally, *CBX4* (Chromobox Homolog 4) and *SLC16A3* (Solute Carrier Family 16, Member 3) genes were selected as epigenetic regulator and target, respectively, in the mouse species, from the study of Goa et *al*. [24]. The gene encoding *SLC38A3* (Solute Carrier Family 38, Member 3) was selected for its protein interaction with *IGF2* (Insulin Like Growth Factor 2) [25].

The objective of the present study was, 1- to investigate the endometrial expression of the 42 genes with parental expression during late gestation and their potential role in fetal maturation and, 2- to unravel the influence of the fetal genome and sex at the feto-maternal interface as potential modulators of the feto-maternal crosstalk during the fetal maturation period (between D90 and D110, gestation length 114 days).

The study allowed us to identify differential endometrial expression between the two breeds for key genes involved in the regulation of angiogenesis, nutrient transport and energy metabolism and highlighted the influence of fetal genotype and sex on endometrial gene expression.

## Results

The mRNA abundance of 42 genes (Additional file 1) was assessed at the maternal interface (endometrium) during late gestation (D90/D110) in two breeds (LW/MS). For that, 88 endometrial samples were collected from LW or MS sows inseminated with mixed LW and MS semen. This protocol allows to correctly address the question of the influence of the fetal genotype on endometrial expression by removing the effect of the uterine environment. The mixed semen consisted of three pairs of males (1 LW + 1 MS). The detailed experimental design is described in Additional file 2. In these conditions, these 88 endometrial samples faced each 88 pure and crossbred fetuses developed together in the same uterine environment (LW or MS). Sample selection includes four to six samples per condition and ranges from two to seven samples per uterus (with at least both genotypes per uterus).

### Endometrial gene expression profile of pregnant sows with different genetics for neonatal survey

The imprinted genes are known to play a critical role in placental development and their expression profiles, in contrast to the endometrium, are well documented. Thus, changes in the expression levels of these genes may influence nutrient transport. Using all the 88 samples, we first investigate whether the expression levels of these genes vary in the endometrium at the end of gestation and between sows of extreme breeds for neonatal survival.

Of the 42 genes, 39 were found to be expressed and profiled in the endometrium. The expression of *ASCL2* (Achaete-Scute Family BHLH Transcription Factor 2), *PHLDA2* (Pleckstrin Homology Like Domain Family A Member 2) and *MESP1* (Mesoderm Posterior BHLH Transcription Factor 1) genes were not detected. Table 1 describes the 22 genes that showed global significant differences for days of gestation (D) x breeds, additive effects, interaction effect between D and breeds, only breeds or only days of gestation (D).

**Table 1:** Global effects of breeds and days of gestation on endometrial gene expression

GENE	Full model	Additive model	D: Breed	D (D110/D90)		Breed (MS/LW)	
	FDR	FDR	FDR	FDR	FC	FDR	FC
GRB10	3.10 10^-03^	2.20 10^-03^				1.40 10^-03^	1.33
DHCR7	2.70 10^-03^	2.80 10^-03^		9.60 10^-04^	0.45		
MEST	8.10 10^-04^	1.20 10^-03^		4.80 10^-04^	0.34		
PEG10	1.40 10^-02^	1.00 10^-02^		4.50 10^-03^	0.47		
TFPI2	3.60 10^-02^	1.70 10^-02^		3.60 10^-02^	1.30		
SLC38A4	2.70 10^-02^	1.20 10^-02^				3.30 10^-02^	0.833
SLC22A3	3.60 10^-02^	1.90 10^-02^		1.30 10^-02^	0.73		
IFG2R	2.50 10^-02^		0.095				
NNAT	2.30 10^-02^	1.20 10^-02^				9.61 10^-03^	1.29
DCN	1.00 10^-02^	3.80 10^-03^				2.40 10^-03^	1.42
QPCT	1.80 10^-02^	4.40 10^-02^		2.80 10^-02^	1.26		
AMPD3	2.30 10^-04^	1.00 10^-04^		5.20 10^-03^	0.83	3.60 10^-04^	1.3
NDP	9.00 10^-04^	1.00 10^-02^	0.055	5.20 10^-03^	0.61		
NES	2.50 10^-02^	1.10 10^-02^		5.20 10^-03^	0.79	2.40 10^-02^	1.21
ASNS		4.50 10^-02^		5.20 10^-03^	1.1		
CITED1	9.10 10^-04^	2.20 10^-03^		5.20 10^-03^	0.38		
TSPAN33	3.50 10^-03^	2.40 10^-03^				1.60 10^-03^	0.72
CDKN1C	1.40 10^-02^	8.80 10^-03^		3.40 10^-03^	0.70		
CREM	3.00 10^-02^	1.40 10^-02^				2.70 10^-02^	0.86
ASPSCR1	2.70 10^-02^	1.20 10^-02^		2.00 10^-02^	0.76		
H19	2.20 10^-03^	2.80 10^-03^		9.60 10^-04^	0.45		
SLC38A2	3.20 10^-02^	1.40 10^-02^				2.80 10^-02^	0.83

The significant differential expressions of the 22 genes are summarized according to whether they are significant for the full model (corresponding to model 1: D x breed effects), the additive model (model 2: D + breed effects), the interaction model (between D and breeds), the gestation days model (model 3: D) and the breed model (model 4: MS/LW). The complete sampling design was used.

The fold change (FC) and the Benjamini-Hochberg corrected *p*-value (FDR) are given for each significant differential gene expression.

D: day of gestation; Breed (MS/LW): comparison between Meishan vs Large White breed; D (D110/D90): comparison between 110 and 90 days of gestation; D: Breed: interaction between days of gestation and breeds

The expression of 14 genes varied between D90 and D110 (FDR < 0.05; Table 1). We observed a significant decrease in the expression at D110 compared to D90 for 11 genes (*PEG10* (Paternally expressed 10), *AMPD3* (Adenosine Monophosphate Deaminase 3), *NDP* (Norrin Cystine Knot Growth Factor NDP), NES (Nestin), *CITED1*(Cbp/P300 Interacting Transactivator With Glu/Asp Rich Carboxy-Terminal Domain 1), *CDKN1C* (Cyclin Dependent Kinase Inhibitor 1C), *ASPSCR1* (Alveolar soft part sarcoma chromosome region, candidate 1), *H19, DHCR7* (7-Dehydrocholesterol Reductase), *MEST*, *SLC22A3* (Solute Carrier Family 22 Member 3)) and the over-expression of three genes (*TFPI2 *(Tissue Factor Pathway Inhibitor 2), *QPCT* (Glutaminyl-Peptide Cyclotransferase) and *ASNS (*Asparagine Synthetase (Glutamine-Hydrolyzing))). The expression of nine genes differed between the two breeds regardless of the day of gestation (FDR<0.05; Table 1). Five genes were up-regulated in MS compared to LW (*GRB10* (Growth Factor Receptor Bound Protein 10), *NNAT* (Neuronatin), *DCN* (Decorin), *AMPD3*, *NES*) and four genes were down-regulated (*SLC38A2* (Solute Carrier Family 38 Member 2)*, SCL38A4* (Solute Carrier Family 38 Member 4)*, TSPAN33* (Tetraspanin 33) and *CREM* (CAMP Responsive Element Modulator)). Last, the analysis identified *NDP* and *IGF2R* (Insulin Like Growth Factor 2 Receptor) genes that tended to show relative expression that varied between breeds, depending on the days of gestation (FDR = 0.055 and 0.095, respectively; interaction effect, Table 1). For these two genes, we observed a significant lower expression at D90 for *IGF2R* and a significant higher expression at D110 for *NDP* in MS compared to LW (Table 2, Additional file 3).

**Table 2:** Differential gene expression according to gestation days and breed subsets

GENE	MS/LW D90		MS /LW D110		D110/D90 LW		D110/D90 MS	
	FDR	FC	FDR	FC	FDR	FC	FDR	FC
GRB10	1.300 10^-02^	1.3	6.260 10-02	1.37				
DHCR7					3.000 10^-03^	0.36		
MEST					1.100 10^-03^	0.25		
PEG10					1.600 10^-02^	0.44		
TFPI2					4.800 10^-02^	1.33		
SLC38A4								
SLC22A3					3.900 10^-02^	0.66		
IFG2R	1.300 10^-02^	0.78			1.800 10^-02^	0.83		
NNAT			3.200 10^-02^	1.41				
DCN	1.300 10^-02^	1.43	8.800 10^-02^	1.40				
QPCT	2.400 10^-02^	0.84					8.500 10^-03^	1.46
AMPD3	2.900 10^-03^	1.34	7.600 10^-02^	1.25			3.400 10^-02^	0.81
NDP			4.000 10^-02^	1.84	1.100 10^-03^	0.36		
NES								
ASNS								
CITED1					1.100 10^-03^	0.29		
TSPAN33	1.260 10^-02^	0.69	7.900 10^-02^	0.74				
CDKN1C					1.600 10^-02^	0.66		
CREM			7.800 10^-02^	0.83				
ASPSCR1					4.000 10^-02^	0.73		
H19					2.200 10^-03^	0.34		
SLC38A2	3.400 10^-02^	0.82	9.200 10^-02^	0.84				

The significant differential expressions of the 22 genes are summarized according to whether they are significant between breeds at each day of gestation (model 3) and between days of gestation for each breed (model 4)

The fold change (FC) and Benjamini-Hochberg corrected *p*-value are given for each significant differential gene expression (FDR<0.1)

**Fig. 1:**
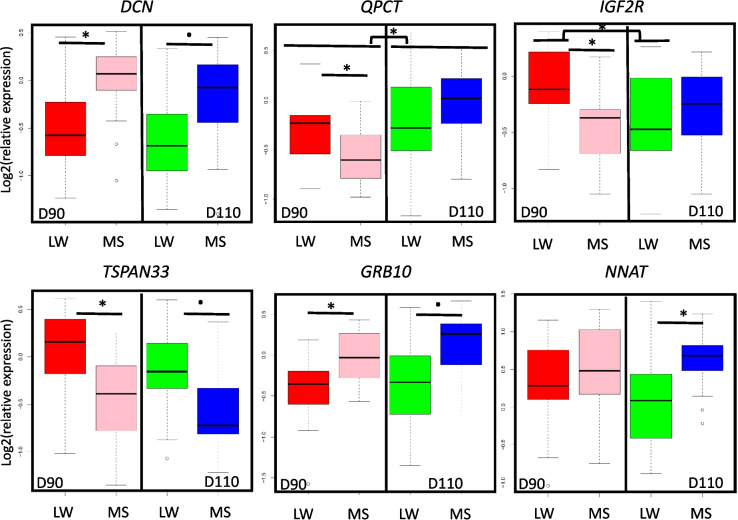
Endometrial gene expression profiles of *DCN*, *QPCT*, *IGF2R*, *TSPAN33*, *CREM*, *NNAT. *Relative quantification throughout the end of gestation at day 90 (D90) or at day 110 (D110) and in two breeds (Large White (LW); Meishan (MS)). The Y-axis corresponds to the relative endometrial expression normalized with three reference genes (*RPL32*, *eEF1* and *RPL19*). The X-axis corresponds to the two breeds and the two days of gestation. ^•^FDR<0.1; * FDR<0.05

Subsequent analyses focused on the 22 DEG (differentially expressed genes) from the full and additive models (FDR <0.05). Table 2 shows that at D90, the expression of four genes (*IGF2R*, *QPCT*, *TSPAN33* (Tetraspanin 33), *SLC38A2*) decreased and the expression of three genes (*GRB10*, *DCN* and *AMPD3*) increased in MS compared to LW. At D110, three genes were down-regulated (*TSPAN33*, *CREM* and *SLC38A2*) and five genes were up-regulated in MS compared to LW (*GRB10*, *NNAT*, *DCN*, *AMPD3* and *NDP*). The differential expression of five genes between breeds was maintained at each day of gestation (*GRB10*, *DCN*, *AMPD3*, *TSPAN33* and *SLC38A2*). Finally, the change in gene expression at the end of gestation (D110/D90) mainly affected the LW breed, i.e. 11 genes in LW versus 2 in MS (*QPCT*, *AMPD3*). Figure 1 illustrates these results with the expression profiles of *DCN*, *QPCT*, *IGF2R*, *TSPAN33*, *GRB10* and *NNAT*, which will be discussed below. At D90, we visualized a significant increase in *DCN* and *GRB10* gene expression and a decrease in gene expression for *QPCT*, *IGF2R* and *TSPAN33* in MS endometrium compared to LW. At D110, *TSPAN33* gene tends to be less expressed in MS endometrium than in LW, but *DCN*, *GRB10* and *NNAT* are more expressed in MS endometrium.

**Fig. 2:**
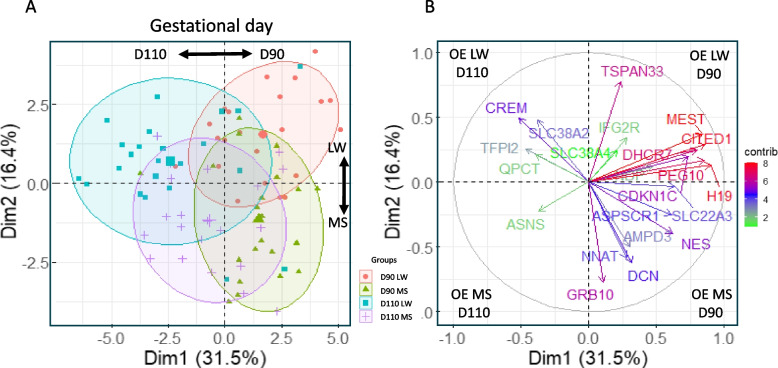
Principal component analysis (PCA). PCA was performed on the 22 Differentially expressed genes (DEG). A- Two-dimensional principal subspace for the DEG data. At D90, samples from LW sow and MS sows are represented by red circles and green triangles, respectively. At D110, samples from LW sows and MS sows are represented by blue squares and purple crosses, respectively. The ellipses have been added to gather the groups (ellipse level=0.75). B- Correlation circle. The plot visualizes the genes correlated with the first two principal components. OE: over-expression

We carried out principal component analysis from the 22 regulated genes to confirm the relevance of the results. This descriptive analysis showed a clear separation between gestation days and sow breeds (Fig. 2A). The first axis explained 31.5% of the expression variability and discriminated the endometrium according to gestation days (D90/D110). The second axis explained 16.4% of the expression variability and discriminated the endometrium according to sow breed (LW/MS). Finally, the ellipses of the PCA plot visualized a greater dispersion of the expression in the LW endometrium than in the MS endometrium at D110. The correlation circle shows the genes that contribute most to the determination of the axis. The first axis of the correlation circle (Fig. 2B) confirmed the opposite regulation pattern reported in Table 1 with *MEST*, *DHCR7* (7-Dehydrocholesterol Reductase), *CITED1*, *PEG10* and *H19* over-expressed genes at D90 and *TFPI2*, *ASNS*, *QPCT*, *CREM* and *SLC38A2* over-expressed genes at D110. The second axis of the correlation plot separated *TSPAN33*, *IGF2R* and *SLC38A2* over-expressed genes in LW to *NNAT*, *DCN* and *GRB10* over-expressed genes in MS. Finally, in this study LW endometrium can be characterized by an over-expression at D90 of *TSPAN33*, *DHCR7* and *MEST* genes and at D110 of *SLC38A2* and *CREM* genes. MS endometrium may be characterized by a strong over-expression of *DCN* and *GRB10* genes compared to LW endometrium.

### Associations between endometrium gene expression and fetal phenotypes as indicators of fetal maturation

To determine whether the expression of the 22 genes are important at the feto-maternal interface and may influence fetal maturation processes, we examined the relationship between the 22 DEGs and three sets of fetal phenotypes (plasma parameters and placental and fetal biometric measures) available for all the fetuses and known to vary during the neonatal period. Six of the fourteen fetal plasma parameters previously analyzed by Yao et *al*. [26] were selected as indicators of metabolic changes that occur late in gestation (glucose, lactate, fructose, albumin, IGF1, cortisol). Four fetal (body weight, body length, body mass index (BMI) and ponderal index (PI) and four placental (weight, area, width and efficiency) biometric measures were also selected as indicators of fetal growth and maturity. Given the large changes in gene expression at the end of gestation, analyses were performed at each stage of gestation to emphasize stage specificity.

**Fig. 3:**
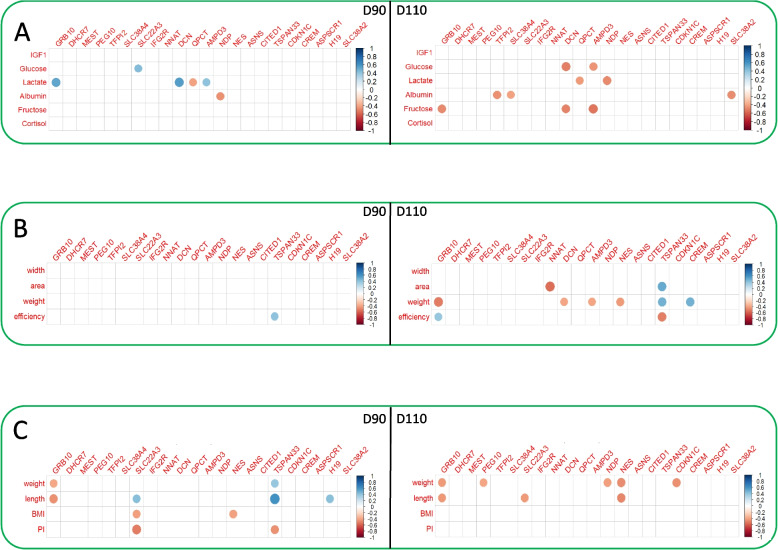
Correlation matrix between DEG and fetal phenotypes. Spearman correlation matrix of the normalized relative expression of the 22 DEG and fetal phenotypes: A- Fetal plasma parameters: IGF1, glucose, lactate, albumin, fructose and cortisol B- Placental biometric measures: placental width, placental area, weight and efficiency (fetal weight (g)/ placental weight (g)), C- Fetal biometric measures: weight, length, BMI and PI. The legend shows the color gradient indicating the direction, positive (blue) and negative (red), of the correlation, and the magnitude of the correlation coefficient. Only the significant correlations (with a *P*-value < 0.05) are plotted.

**Fig. 4:**
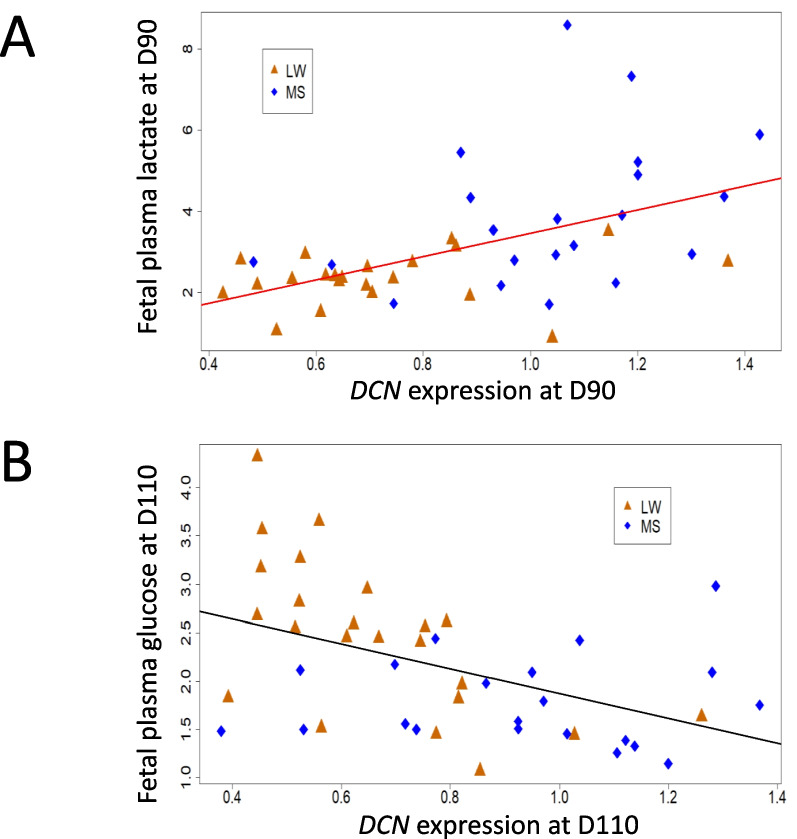
Plot correlating the endometrial *DCN* expression with fetal plasma parameters. A- Correlation between *DCN* expression and plasma lactate level at D90. B- Correlation between *DCN* expression and plasma glucose level at D110. The X-axis corresponds to the relative expression of *DCN* in endometrium. The Y-axis corresponds to the lactate or glucose level in fetal plasma. The regression line was calculated using the D90 data for correlation with lactate or D110 data for correlation with glucose.

First, we carried out a correlation analysis to assess the association between the expression of the 22 DEGs in endometrium and the phenotypes of their respective fetuses. The correlation matrices are shown in Figure 3. At D90, the analysis identified six (*GRB10*, *SLC22A3*, *DCN*, *QPCT*, *AMPD3*, *NDP*), one (*TSPAN33*) and five genes (*GRB10*, *SLC22A3*, *NES*, *TSPAN33, H19*) with significant correlations (*p*<0.01) for fetal plasma parameters, placental and fetal biometric measurements (Fig 3A, 3B and 3C, respectively). At D110, the significant correlations concerned the expression of eight (e.g. *SLC38A4*, *SLC38A2, AMPD3*), seven (e.g. *GRB10, TSPAN33*) and six (e.g. *GRB10, NES*) genes (Fig 3A, 3B and 3C, respectively). This analysis highlighted three phenotypes with the highest number of gene expression correlations. Lactate concentration in fetal plasma correlated with the expression of five genes (Figure 3A: *GRB10*, *DCN*, *QPCT*, *AMPD3* at D90 and *QPCT*, *NDP* at D110). Placental weight correlated with the expression of six genes at D110 (Figure 3B; *GRB10*, *DCN*, *AMPD3, NES, TSPAN33, CREM*). Fetal body weight correlated with the expression of six genes (Fig. 3C: *GRB10*, *TSPAN33* at D90; *GRB10*, *PEG10*, *NDP, NES, CDKN1C* at D110). Taken together, *SCL22A3* and *TSPAN33* had the highest number of phenotypic correlations. The expression of the *SLC22A3* gene correlates with three fetal biometric measures (body length, BMI, PI) (Fig. 3C, D90) and the *TSPAN33* gene correlates with six phenotypic variables derived from fetal biometric measures (weight, length and PI) at D90 (Fig. 3C) and placental biometric measures (area, weight and efficiency) at D110 (Fig. 3B). In particular, the expression of *DCN*, identified above with significant interaction effects (effect of day of gestation as a function of breed: Table 1), shows correlations with fetal plasma lactate at D90 (Fig. 4A) and glucose at D110 (Fig. 4B). Figure 4 shows a progressive separation of the two maternal genotypes along the regression line. At D90, endometrial DCN expression and plasma lactate levels were lower in LW than in MS (Fig. 4A), but at D110, plasma glucose (Fig. 4B) and fructose (not shown) are higher in LW than in MS.

**Fig. 5:**
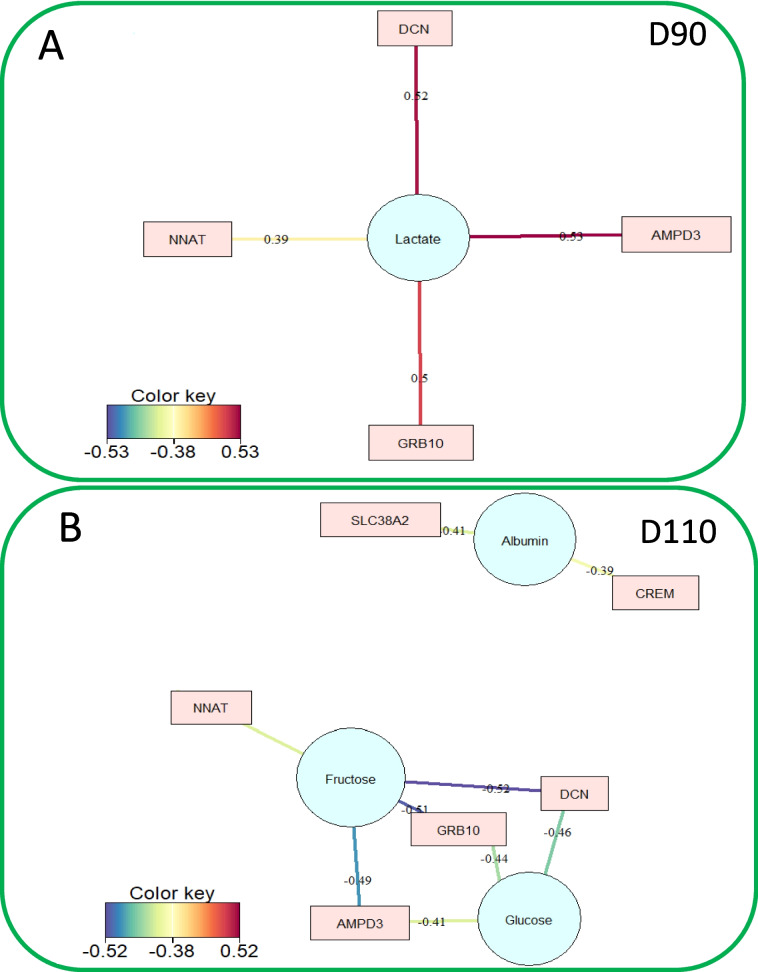
Correlation between DEG and plasma parameters using bipartite networks. Correlation networks were developed for each gestational day between the 22 DEG with: A- Plasma parameters at D90, B- Plasma parameters at D110. The legend depicts the color gradient indicating the direction, positive (blue) and negative (red) of the correlation, and the magnitude of correlation coefficient. A cutoff of 0.38 was applied to the network.

**Fig. 6:**
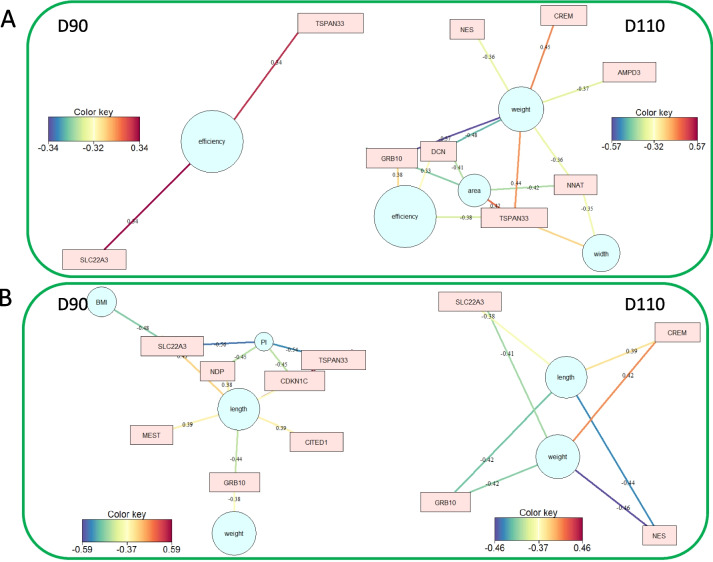
Correlation between DEG, placental and fetal biometric measures using bipartite networks. Correlation networks were developed for each gestational day between the 22 DEG using: A- Placental biometric measures at D90 and D110, B- Fetal biometric measures at D90 and D110. The legend depicts the color gradient indicating the direction, positive (blue) and negative (red) of the correlation, and the magnitude of correlation coefficient. A cutoff of 0.32 and 0.37 was applied to the placental and fetal networks, respectively.

Partial least squares regression analyses (PLS) were used to explore the best correlation networks and attempt to explain the relationship between gene expression and the three groups of fetal phenotypes. At D90, lactate (Fig. 5A, correlation threshold |0.38|), placental efficiency (Fig. 6A D90, correlation threshold |0.32|), body length, and PI (Fig. 6B D90, correlation threshold |0.37|) were the phenotypic variables that had the highest correlations with DEG expression. At D110, fetal plasma metabolites generated two networks with four genes correlated with fructose and glucose (*DCN*, *GRB10*, *NNAT*, *AMPD3*) and two genes correlated with albumin (Fig. 5B; *SLC38A2*, *CREM*). Placental biometric measures generated a network of seven genes (Fig. 6A D110) that confirmed and complemented the previous analysis. Particularly, placental weight was associated with the expression of the seven genes with positive (*CREM* (*r*=0.45) and *TSPAN33* (*r*=0.44)) or negative (*GRB10* (*r*=-0.57), *NES* (*r*=-0.36), *DCN* (*r*=-0.48), *AMPD3* (*r*=-0.37), *NNAT* (*r*=-0.36)) correlations. The fetal biometric measures had more correlations with DEG expression at D90 than at D110, generating a network of seven genes (Fig. 6B). At D90, body length was the phenotype most highly correlated with gene expression (*GRB10*, *CITED1*, *MEST*, *SLC22A3*, *CDKN1C*, *TSPAN33*). At D110, fetal weight is associated with the expression of four genes (Fig. 6B *GRB10*, *NES*, *CREM*, *SLC22A3*).

### Fetal sex and genotype interact to affect gene expression in pig endometrium

The genetic protocol (see Methods section) produced in each pregnant sow, during the same gestation, both purebred (LL or MM) and crossbred fetuses (LM from MS sows and ML from LW sows) together. As a result, each piece of endometrium from a pregnant sow was associated with a purebred or crossbred fetus and with male or female fetuses and could be used to address the question of the influence of fetal sex and genotype on endometrial expression in a same uterine environment. The D110 subset was not evaluated due to an unbalanced distribution of fetal genotype and sex within the sow. At D90, six of the 22 genes selected above were identified with significant differential expression (FDR< 0.05) for the full model (fetal genotype * sex), and corresponding to either additive effects (fetal genotype + sex: GRB10, AMPD3, H19), interaction effect (fetal genotype: sex: DHCR7, MEST, CITED1), or genotype effect only (GRB10, AMPD3). The results are shown in Table 3.

**Table 3:** Statistics of differentially expressed genes as a function of fetal genotype and/or sex at D90

Models	Fetal genotype * sex	Fetal genotype + sex	Fetal genotype : sex	Fetal genotype
GRB10	0.0176	0.0152		0.00933
DHCR7	0.00354		0.00396	
MEST	0.0273		0.0366	
AMPD3	0.00261	0.0038		0.00348
CITED1	0.00366		0.00524	
H19	0.05		0.0366	

The Benjamini-Hochberg corrected *p*-values of the six genes are summarized according to whether they are significant (FDR<0.05) for the full model (model 5: fetal genotype, sex and their interactions), the additive model (model 6: fetal genotype and sex), the interaction model (fetal genotype: sex), or the genotype-only model 7 at D90.

### Fetal sex affects gene expression in pig endometrium at D90

Finally, a Tukey post hoc test was performed on the 6 genes from Table 3 to determine the significant differential expression associated with fetal sex at D90 in each breed subset.

**Fig. 7:**
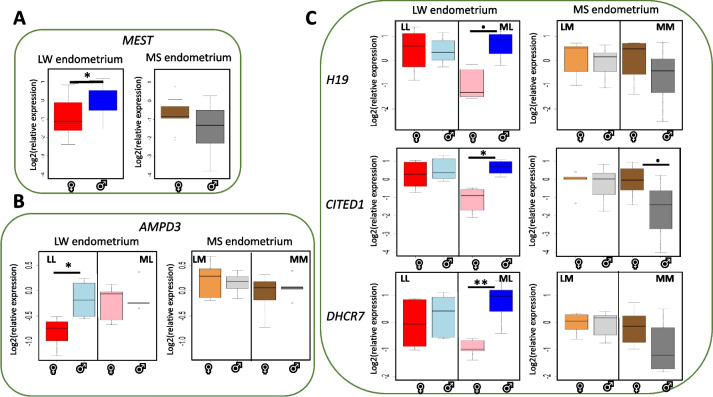
Box-plot of the expression of endometrial genes in relation to the sex of the fetus at D90. A: Fetal sex affects gene expression of *MEST* in the porcine LW endometrium regardless of the genotype of the associated fetus. B- Fetal sex affects gene expression of *AMPD3* in the porcine LW endometrium associated with purebred fetuses (LL). C- Fetal sex affects gene expression of *H19, CITED1 and DHCR7* in the porcine LW endometrium associated with crossbred fetuses (ML). *CITED1* gene expression tends to be influenced by fetal sex in MS endometrium associated with purebred fetus (MM). Gene expression is log2 transformed. From the Large White sows (LW) - LL: LWxLW purebred fetuses - ML: MSxLW crossbred fetus. From the Meishan sows (MS) - MM: MSxMS purebred fetuses – LM: LWxMS crossbred fetus.  ^•^FDR<0.1; * FDR<0.05, ** FDR<0.01

Figure 7 shows the significant comparisons between sex. In LW, the expression of *MEST* varied when the endometrium was associated with male or female, irrespective of the fetal genotypes (Fig. 7A). We showed a differential expression function of fetal sex for the *AMPD3* gene in the endometrium associated with LL fetuses (LWxLW purebred fetuses) (Fig. 7B) and for three genes (H19, *CITED*1, *DHCR7*) in the endometrium associated with ML fetuses (MSxLW crossbred fetuses from LW sows) (Fig. 7C). The gene expression of these five genes (*MEST*, AMPD3, *CITED1, DHCR7*, *H19*) was decreased in endometrial samples associated with female fetuses compared to male fetuses. In contrast, in MS, *CITED1* expression tended to increase in endometrium associated with female MS compared to male endometrium (FDR= 0.086). No significant difference was observed in MS endometrium associated with crossbreed (LM).

### Fetal genome affects gene expression in pig endometrium at D90

**Fig 8:**
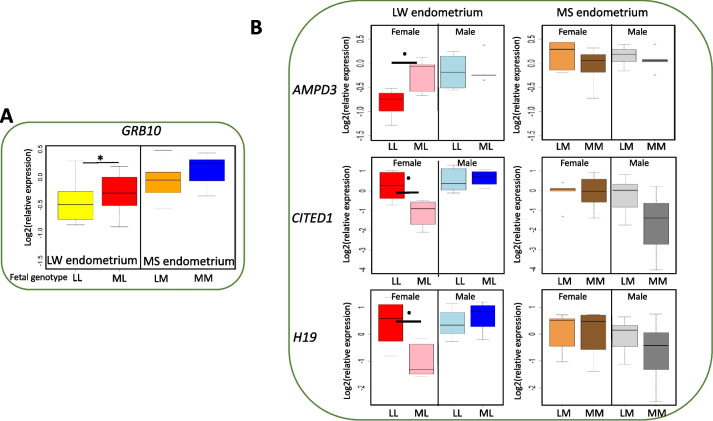
Box-plot of the endometrial gene expression in relation to the genotype of the fetus at D90. A- Fetal genotype affects the expression of *GRB10* in the LW endometrium. B- *AMPD3*, *CITED1* and *H19* endometrial gene expression in LW tends to vary as a function of the female's fetal genotype. Gene expression is log2 transformed. From the Large White sows (LW) - LL: LWxLW purebred fetuses - ML: MSxLW crossbred fetus. From the Meishan sows (MS) - MM: MSxMS purebred fetuses – LM: LWxMS crossbred fetus. FDR<0.1; * FDR<0.05

Fig. 8 shows the significant comparisons between fetal genotypes at D90 in each breed subset and function of sex for the 6 genes from Table 3 (Tukey post hoc test). At D90, fetal genome influenced the expression of *GRB10*, *AMPD*3, *CITED1* and *H19* genes in LW endometrium (Fig. 8).

The expression of *GRB10* was globally influenced by the fetal genome. The expression of three other genes (*AMPD3*, *CITED1*, *H19*) was exclusively influenced by the female fetal genome. The expression of *AMPD3* increased in the endometrium associated with female fetuses inheriting a MS paternal genome compared to female fetuses with a LW paternal genome. In contrast, the expression of *CITED1* and *H19* decreased. No statistically significant associations between fetal genotype and endometrial expression were observed in MS endometrium.

## Discussion

Supporting pregnancy requires a fine balance between the nutritional requirements for optimal fetal growth and those of the sow. Therefore, it is of paramount importance to determine how the feto-maternal system functions and could be optimized to increase piglet maturity and survival at birth. In the uterus, the expression of endometrial genes changes dynamically depending on the day of gestation [12, 27] and is affected by maternal hormones and the conceptus [28]. Many studies have investigated the expression and function of endometrial genes during the implantation window [29], but few have described the end of pregnancy. On the other hand, the placenta regulates feto-maternal allocation of resource by adapting its phenotype through epigenetic mechanisms [30]. Most imprinted genes are expressed in the placenta and play a critical role in the regulation of nutrient supply [31]. To date, these genes are poorly described in the endometrium.

The current study showed for the first time that the expression of some genes, including imprinted genes (e.g. *MEST*, *CITED1*, *CDKN1C*, *H19*, *SLC22A3*, *PEG10*), changed in the endometrium at the end of gestation and between two breeds: LW vs. MS, showing high and low neonatal piglet mortality, respectively. In response to fetal demand, endometrial strategy differs between MS and LW sows to optimize resource allocation and fetal maturity. The timing of gene expression changes during gestation may play a critical role in endometrial remodelling and in placental and fetal development [12]. The higher change in gene expression in LW during late gestation (11 genes) compared to MS (two genes) suggests that more molecular events are occurring in the LW endometrium at this stage compared to MS, possibly reflecting delayed or altered progression of endometrial processes in LW compared to MS sows such as the angiogenic process discussed below, which may result in LW piglets being less mature at birth than MS piglets. More extensive gene expression studies are needed to confirm this initial observation. Finally, our study demonstrated that fetal sex and genotype influence the expression of key genes in the porcine endometrium at D90 of gestation, a critical stage that marks the onset of fetal maturation processes in pigs. Particularly, fetal sex affects the expression of imprinted genes such as *H19*, known for its role in regulating fetal growth and nutrient transfer through its interaction with IGF2.

### Involvement of angiogenic process at the end of gestation

Maximal fetal and placental growth occurs toward the end of gestation, resulting in a nutritional burden on the mother. Fourteen of the 39 genes tested were found to be differentially expressed in the endometrium between 90 and 110 days of gestation (D90-D110), illustrating the final push to ensure a steady supply of nutrients to the placenta and fetus. For example, *ASNS* gene expression increased at D110. Several studies have shown that in response to intracellular amino acid, glucose or protein deprivation, *ASNS* transcription is activated via the amino acid response (AAR) pathway to increase protein synthesis [32, 33]. A maternal low-protein diet is able to activate the placental AAR pathway and may act as a programming signal to adapt the fetus to its postnatal environment [34]. Therefore, in the endometrium in late gestation, this *ASNS* over-expression may be associated with amino acid deficiency or imbalance.

Other DEGs for gestation day showed reduced expression at D110 and were involved in growth, such as *CDKN1C,* or cholesterol metabolism, such as *DHCR7,* the final enzyme in the cholesterol biosynthetic pathway. These differential expressions may reflect insufficient nutrient availability to meet the overall metabolic needs of sows during late gestation [35], resulting in an increase in their catabolic status at D110 [36].

As previously shown by Udagawa et *al.* in human placenta [37, 38], we highlighted the upregulation of *TFPI2* at D110 in pig endometrium, consistent with its potential role in maintaining microvasculature in the feto-maternal blood system. Indeed, *TFPI2* is a critical gene that regulates the coagulation pathways and the angiogenesis process [39]. It belongs to the TFPI (Tissue Factor Pathway Inhibitor) family, which inhibits the tissue factor (TF) pathway, a key activator of the coagulation cascade, resulting in an efficient hemostatic mechanism. Changes in the coagulation system during gestation have been reported to be adaptive [40]. Lockwood *et al* [41] described a procoagulant state for the maternal decidua to prevent maternal bleeding at the time of delivery. The authors also mentioned a hypocoagulant state in the intervillous space of the maternal-fetal interface to ensure extravascular laminar flow of maternal blood. Udagawa et *al.* [37] suggested that TFPI2 regulates placental perfusion maintaining intervillous blood flow around the chorionic villi to support adequate nutrient exchange between the fetus and maternal blood. Our results reinforce the importance of the hemostatic balance in endometrium up to D110 to support nutrient demand.

The literature also suggested a pivotal role for *MEST* in placental development, including angiogenesis [42] and invasion of extravillous trophoblast cells into the maternal uterine decidua [43]. *Mest*-deficient mice have fetal growth restriction and increased perinatal and postnatal lethality [43]. As in the placenta, downregulation of *MEST* at D110 in the endometrium may be related to the angiogenic process.

Finally, the *SLC22A3* gene*,* under-expressed at D110, encodes a polyspecific organic cation transporter that plays an important role in decidualization, implantation, immune modulation and inflammation [44]. In the rat, SLC22A3 is an essential component of feto-placental serotonin homeostasis and protects the placenta from local vasoconstrictive effects of serotonin [45]. It is therefore involved in the regulation of blood flow and capillary permeability, but also in related pathways such as the transport of glucose and other sugars.

### Differences in energy resources management between MS and LW sows

The expressed DEGs between the two breeds are involved in vascular network, nutrient transport and energy storage.

Genes involved in endometrial vascularization: Vascular development and vascular permeability are important for nutrient exchange at the feto-maternal interface. Previously published data reported increased endometrial vascular density in the MS compared to the Yorkshire breed at D110 [46]. A comparison between MS and Duroc endometrial gene expression at D72 identified several genes involved in blood vessel development that were not present at D49. Their results suggest increased endometrial angiogenesis in MS, which may explain the denser vascularization observed in MS compared to commercial breeds (LW, Duroc ) [47]. Consistent with these studies, our results also highlighted three DEGs between the two breeds that are associated with angiogenesis (*NDP*, *TSPAN33* and *DCN*). For example, Luhmann *et al.* [48] first identified *ndp* (Norrin Cystine Knot Growth Factor) gene expression in the mouse uterus and decidua and postulated that the *Ndp* homologous −/− female mice had embryonic loss due to the inability of their uterus to support fetal development. Their data provide strong evidence for defective vascular differentiation and decidualization during implantation.

The *TSPAN33* (Tetraspanin 33) gene is a member of the tetraspanin family. This gene family mediates the regulation of signal transduction events and positively regulates the maturation and trafficking of the transmembrane metalloprotease ADAM10 [49]. Adams and tetraspanin proteins are involved in the processes of reproduction and feto-maternal crosstalk. For example, ADAM10 and TSPAN6 (another member of the tetraspanin family) have been identified in the cargo of exosomes from cultured bovine endometrial stromal cells (ICAR cells) [50]. Furthermore, *ADAM10* expression was significantly increased in preeclamptic placental explants compared to normal placentas, which is thought to induce the release of soluble vascular endothelial growth factor receptor-1 (sFlt-1) [51, 52], followed by a cascade of endothelial dysfunction. sFlt-1 is an anti-angiogenic molecule produced by trophoblasts in preeclamptic placentas and is recognized as a blood biomarker in preeclamptic mothers.

DCN (Decorin) is a multifunctional molecule that regulates cell adhesion, migration and invasion. DCN actions at the fetal-maternal interface include inhibition of placental invasion of the pregnant uterus and uterine arterial remodeling. Lala *et al* [53] reviewed its role and reported that *DCN* over-expression in the decidua is associated with pre-eclamptic mothers and may up-regulated the expression of *sFlt-1* already mentioned above [54]. The under expression of *DCN* in LW endometrium compared to MS at D90 suggested less vascularization remodeling in LW endometrium.

These DEGs indicated differences between MS and LW in endometrial angiogenic regulation at the end of gestation (vascularization, blood flow, perfusion); they suggested less nutrient perfusion from the LW endometrium to the fetus compared to MS, resulting in both lower maturity of their piglets at birth and an increase in perinatal mortality.

Genes involved in nutrient transport and energy metabolism: Maternal-fetal exchange is mediated in part by imprinted genes. The metabolic nutrients for the fetus are glucose, fatty acids and amino acids and are thought to be actively transported across the maternal-fetal barrier, i.e. across three maternal and three fetal layers in the porcine diffuse placenta. Our study illustrated this endometrial trafficking with the upregulation of some solute carriers in LW compared to MS *i.e*. *SLC38A2* and *SLC38A4*. The *SLC38A2* gene belongs to the System A (alanine-preferring) amino acid transporters and is involved in hyperosmotic stress and endoplasmic reticulum stress. Particularly during starvation of one or more amino acids such as glutamine, *Slc38a2* transcription is transiently enhanced [55]. Its expression also increases during early pregnancy in the equine endometrium [56], and the inhibition of its activity in the rat placenta reduces fetal weight [57]. The *SLC38A4* gene mainly transports cationic and neutral amino acids such as arginine, lysine and alanine. The overexpression of these genes suggested an increase of amino acid transport to the fetus in LW sows compared to MS.

Glucose is a major source of energy required for fetal growth and development. In late gestation, the fetal demand for glucose from the mother has increased dramatically, resulting in abnormal maternal glucose metabolism. During this period, maternal insulin resistance, which is part of the metabolic syndrome of late pregnancy, is particularly elevated [58] and is associated with fetal growth rate [59]. In fact, normal glucose metabolism in the endometrium is essential to support pregnancy [60]. Nevertheless, the endometrium is not able to produce glucose *de novo*. As mentioned above, glucose comes from the maternal circulation and the first step in glucose utilization is its uptake into cells via glucose transmembrane transporters. Glucose is then temporarily stored in the endometrium as the macromolecule glycogen before being used or secreted into the uterine lumen during early gestation [61]. A number of imprinted genes have been found to influence energy homeostasis and some, including *Igf2, H19* and *Grb10*, have been reported to be major regulators of both fetal and placental growth and metabolism, including glucose-regulated metabolism.

There is evidence from the mouse placenta-specific Igf2 deficiency, which results in a restrained placenta, that IGF2 can modulate amino acids and glucose transport in late gestation, in part by regulating the expression of the transporter genes *Slc2a3*, *Slc38a4* and *Slc38a2* [25]. In our study, although *IGF2* gene expression is stable at the end of gestation and between breeds in the endometrium, *IGF2R*, which controls IGF2 activity, is overexpressed in LW at D90 compared to MS. As in the placenta, our results were consistent with a potential role of IGF2R/IGF2 in facilitating the transport of nutrients at the feto-maternal interface, such as glucose and amino acids, to support fetal growth, ensuring efficient transfer of essential nutrients from the maternal circulation to the developing fetus via the placenta.

GRB10 (Growth Factor Receptor Bound Protein 10) is an adaptor protein that interacts with numerous intracellular signaling molecules, including receptor tyrosine kinases, such as EGFR, insulin receptor and IGF1R. Gene knockouts in mice have identified Grb10 as an inhibitor of intracellular signaling pathways regulating growth and metabolism. Charambous et *al.* used *Grb10* deletion on the maternal allele to show that GBR10 restricts embryonic and placental growth essentially through an Igf2-independent pathway [62]*.* Maternally inherited placental Grb10 acts to limit the labyrinthine volume, the nutrient exchange compartment in the placenta, thereby limiting fetal growth and protecting the mother from pregnancy complications. On the mother side, Grb10 dosage may modulate maternal resource allocation and influence reproductive strategies by regulating the balance between offspring number and offspring weight [63]. Our results showed an increase in *GRB10* expression in MS compared to LW endometrium, suggesting a more stringent regulation of nutrient supply by MS sows, in accordance with a lower weight of MS offspring compared to LW [64].

Our study also highlighted the over-expression of *NNAT* (Neuronatin) in MS compared to LW at D110. In the pig, NNAT protein was found in the uterine luminal and glandular epithelium of the placenta [65]. In placenta, *NNAT* mRNA expression differed between MS and Yorkshire at D75. An over-expression of *NNAT* induced intracellular glucose uptake and improved glucose transport *in vitro* through activation of the PI3K-AKT pathway [65, 66]. Consistent with this, our results suggested more glucose trafficking in MS endometrium than LW.

Finally, AMPD3 (Adenosine Monophosphate Deaminase 3) is an AMP deaminase (AMPD family) that catalyzes the irreversible deamination of AMP to IMP and NH3. It controls the content of intracellular adenine nucleotides (ATP, ADP, and AMP), thereby regulating mitochondrial protein synthesis rates and cellular metabolism. AMPD3 is the main protein isoform present in erythrocytes, and its expression can be detected in a wide range of other tissues such as muscle and placenta [67, 68, 69]. The over-expression of *AMPD3* in cultured mouse muscle is able to alter intra- and extracellular metabolome. Its over-expression increased the intracellular glucose, sorbitol, and fructose suggesting an increased glucose flux into the polyol pathway [67] for *de novo* glutamine and alanine synthesis [67].

Taken together, these results suggest an increased nutrient supply to the fetus in LW sows compared to MS sows in late gestation, e.g. greater amino acid transport to the fetus in the LW endometrium, which may compensate for the lower vascular endometrial density [46]. Furthermore, *NNAT* and *AMPD3* gene expression suggests that there is more intracellular glucose in the MS endometrium than in the LW, promoting glycolysis through the alternative polyol pathway to produce fructose. All the enzymes belonging to the polyol and fructose transporters are present at the feto-maternal interface in pigs [70]. The lower intracellular level of glucose in the LW endometrium may be associated with greater transfer to the fetus, consistent with Yao's study showing a tendency for higher blood glucose concentration in LW fetuses compared to MS [31].

### Relationship between endometrium gene expression and fetal phenotypes describing maturation

Correlations and PLS analyses were performed to combine gene expression and fetal phenotypes identified as indicators of fetal maturation and to gain insight into the influence of endometrial gene expression in determining fetal maturation.

Correlations and PLS analyses highlighted a strong negative relationship between the expression of 4 genes (*DCN*, *AMPD3*, *NNAT* and *GRB10*, Fig. 3 and 5) and the abundance of fetal plasmatic fructose and glucose at D110. These correlations reinforce the involvement of these genes in the regulation of carbohydrate metabolism at the feto-maternal interface. Glucose is one of the main molecules supplying the energy needed to maintain endometrial activity, and is transferred from mother to fetus during gestation, providing the energy needed for placental and fetal growth and piglet maturity [71]. The correlations (Fig. 6) suggested that these genes may influence placental phenotypes such as the placental weight at D110.

Furthermore, we identified differential expression between breeds of *TSPAN33* and *DCN*, which are involved in the regulation of endometrial angiogenesis and positively correlated with placental weight at D110. Placental weight has already been reported to be higher in Yorkshire, another commercial breed [72], to compensate for less vascularization. These correlations for genes involved in angiogenesis suggest that endometrial vascularization may also influence placental weight and may participate to maturity at birth.

### Fetal genotype and sex influenced the expression of endometrial genes

We demonstrated for the first time that not only fetal sex but also fetal genotype influenced the expression of key genes in pig endometrium at D90 of gestation.

Fetal sex is known to influence the interaction between the mother, the placenta and the fetus, thus affecting not only the intrauterine life and the health of the pregnant mother, but may also have significant effects on the postnatal life and future health of the mother [73, 74]. If the sexual dimorphism has been described in the placenta [75, 76], little has been documented in the endometrium. Stenhouse et *al.* first described associations between fetal sex and endometrial structure, gene expression associated with angiogenesis, apoptosis, proliferation and endothelial cell branching in pig [20, 21, 77]. Their data suggest that fetal sex may influence the endometrial transcriptome as in the placenta. In the present study, fetal sex affected the expression of five imprinted genes in the LW endometrium at D90, such as the *H19* gene, which is known to negatively regulate the expression of *IGF2* and *IGF2R*, which controls IGF2 activity [78]. Deletion of *H19* results in biallelic expression of *Igf2* and reduced expression of key placental transporter genes (*Slc2a3* and *Slc38a4*), limiting the ability to transfer nutrients to the fetus [79]. These five genes (*AMPD3*, *H19*, *DHCR7*, *MEST*, *CITED1,* Fig. 7) were under-expressed in the LW endometrium when associated with a female fetus compared to a male. Consistent with previous studies [80], these results suggest that male fetuses influence the endometrium to promote angiogenesis and metabolic processes and increase nutrient transfer to accelerate their fetal growth compared to female fetuses. They reinforced the idea that the endometrium adapts differently to the sex of the fetus depending on the breed of the sow (MS or LW) and the fetus genotype.

The current study reported differential endometrial gene expression function of fetal genotype at D90. These results are consistent with other studies that have identified significant differences in endometrial transcript levels between pregnancies depending on the origin of the embryos (nuclear transfer or in vitro fertilized embryos) [17, 81]. The present results suggest that the expression of key genes involved in the regulation of growth (*H19*), vasculature (*CITED1*) and metabolism (*AMPD3*) may be modulated by fetal genotype in the LW endometrium. This modulation was potentially dependent on fetal paternal origin (i.e., paternal MS genotype in this study) and may influence nutrient transfer and fetal maturation. The specific function of these genes in the endometrium remains to be elucidated.

Most interestingly, the study pointed out that the LW endometrium tended to be more sensitive to a crossbred fetal genotype environment than the MS. The LW endometrium was also more sensitive to fetal sex if the endometrium is adjacent to crossbred fetuses.

Further endometrial analysis using high-throughput sequencing technologies and additional samples will be required to decipher the influence of fetal sex and fetal genome at the porcine feto-maternal interface in late gestation.

## Conclusion

The current study revealed 22 genes (DEGs) whose expression that changed in the endometrium at the end of gestation and between two breeds. The main transcriptomic change during late gestation involved genes that participate to the angiogenic processes and refers mainly the LW endometrium, suggesting a delay or an altered progression of endometrial processes in LW compared to MS sows. Nine DEGs were observed between the two extreme breeds for piglet maturity. These genes are involved in the regulation of endometrial angiogenesis, nutrient transport and energy metabolism and showed important relationships with fetal maturation indicators such as ponderal index at D90, fetal blood fructose level and placental weight at D110.

This study provided new evidence for some sexual dimorphism and the influence of fetal genotype on the expression of key imprinted genes in the pregnant endometrium, such as H19, which is known for its role in regulating fetal growth and nutrient transfer through its interaction with IGF2. Our findings illustrated the plasticity of the endometrium and highlighted the importance of elucidating the dialogue between the placenta and the endometrium. In addition, they suggest that the paternal genome, particularly in crossbreeding production systems, may contribute significantly to piglet survival. The processes involved in these genomic interactions at the feto-maternal interface require further investigation.

## Methods

### Ethics statements

#### Experimental design and RNA preparation

Details on animal resources and genetic design can be found in Voillet et *al.* 2014 and 2018 [10, 82]. Briefly, 11 MS and 13 LW sows were inseminated with mixed semen (3 LW and 3 MS boars) at the GenESI experimental farm (https://doi.org/10.15454/1.5572415481185847E12). The mixed semen consisted of three pairs of males (1 LW + 1 MS). Each male pair inseminated an equal number of MS and LW females. Thus, each litter consisted of purebred (LL) and crossbred fetuses (ML) from LW sows or purebred (MM) and crossbred fetuses (LM) from MS sows. The MS and LW breeds are two extreme breeds in terms of fetal maturity and piglet mortality at birth. The Large White (LW) breed is a highly selected breed with high mortality and decreased maturity at birth, whereas the Chinese Meishan (MS) breed produces piglets with extremely low mortality despite being lighter at birth [12, 17]. The litter size is around 14 in both species. All samples in this study are from the experiment previously described by Voillet et *al* and Yao et *al* [10, 26] and were obtained after caesarean delivery and fetal euthanasia (https://data.faang.org/api/fire_api/samples/INRAE_SOP_COLOcATION-tissues_20210817.pdf). After laparotomy of the sow, fetal blood was sampled separately from the umbilical artery and plasma stored at −20 °C. After section of the umbilical cord, the fetuses were euthanized, weighed, sexed, and placental and fetal measurements recorded. They were selected considering their body weight that was the closest to the mean body weight of each litter. These fetuses were genotyped with eight microsatellite markers, to discriminate the crossed breeds from the pure breeds. Gender identification was performed *de visu* because sex was clearly visible without doubt at the end of gestation.

In Pigs, the placenta is epitheliochorial and homogeneous: the trophoblastic and endometrial epithelia are juxtaposed, with no invasion of the trophoblast into the endometrium. For each fetus, a pre-determined area (juxtaposed area, excluding distal parts) of the two adjacent tissues (placenta/endometrium) was dissected, dissociated with forceps, immediately frozen in liquid nitrogen and stored at -80°C. Hence, each endometrium sample was associated with its purebred or crossbred fetus: LWxLW (LL) or MSxLW (ML) from Large White sows (LW) and MSxMS (MM) or LWxMS (LM) from Meishan sows (MS). Finally, 88 endometrial samples from two breeds (13 LW and 11 MS) that juxtaposed fetuses of four fetal genotypes and two sexes were selected at 90 and 110 days of gestation; (birth at 114 days), ensuring genotype and sex balance per sow. The number of replicates per condition (4 to 6) is given in Additional file 2. The sampling selection varies between two and seven samples per uterus. Briefly, the 22 LW and 22 MS endometrial samples were collected at D90 from seven LW and six MS sows. They were divided into six replicates for each group of males and females in the LL and MM fetal genotypes and five replicates for each group of males and females in the ML and LM fetal genotypes. At D110, the 23 LW and 21 MS endometrial samples were collected from six LW and five MS sows. They were divided into six replicates for the male and female groups in LL fetuses, six/five replicates for each group of males and females in ML and LM fetal genotypes, and six/four replicates for the male and female groups in MM fetuses.

### RNA extraction

Total RNA was isolated from each of the 88 samples. Briefly, tissue samples were disrupted, homogenized, and ground to a fine powder by rapid agitation for 30 s-1 min in a liquid-nitrogen cooled grinder with stainless steel beads with a Retsch MM400 instrument at 30 Hz. An aliquot of 80-100 mg of the fine powder was then processed for total RNA isolation and purification using Trizol (Invitrogen, France) and Nucleospin RNA II kit (Macherey-Nagel, France) according to the manufacturer's instructions. The method included a DNase digestion step to remove contaminating DNA. Extracted total RNA was eluted in 40 *μl* RNase-free water and stored at -80°C. RNA quality and concentration were verified using an Agilent 2100 bioanalyzer (RNA solutions and RNA 6000 Nano Lab-Chip Kit, Agilent Technologies France, Massy, France).

### Quantitative real time RT-PCR analysis for gene expression

Gene primers were designed from porcine genes considering the intron-exon organization from the Ensembl database. Gene primers were preferentially designed in the 3′ UTR (last 1000 base pairs) or the exons using LightCycler Probe Design2 software (Roche Diagnostics). The primer pairs were confirmed using Primer3 (http://frodo.wi.mit.edu/primer3/). Sequences are available in Supplementary Data 4.

RNA samples were reverse transcribed from 2 *μg* as previously described [83]. The resulting cDNA samples were completed to 100 *μl*. The assay for each gene consisted of four/six replicates per genotype, sex and gestational day, calibrator sample, a serial diluted pool and negative controls.

The expression of the 42 genes was analyzed in duplicate using 96.96 Dynamic Array™ IFCs and the BioMark™ HD System from Fluidigm. Two specific target amplifications (STA) were performed on the 94 cDNA at 5ng/µl (88 endometrial cDNA samples, a calibrator sample (pool of all RNA samples) and a 5-serial dilution of the calibrator sample (for calculation of PCR amplification efficiency) in 96-well PCR according to the manufacturer's recommendations. As previously described [83], a 14-cycle STA treated with Exonuclease I was performed, diluted and transferred to the BioMark™ HD for final STA. Data were then analyzed using the Fluidigm Digital PCR Analysis software using the Linear (Derivative) Baseline Correction method, the User (Global) Ct Threshold method with the threshold set at 0.01. The Fluidigm Digital PCR Analysis software determines the threshold cycle (Ct) of each sample. PCR amplification efficiency was determined specifically for each gene from fluorescence data using LinRegPCR version 2012.2 [84]. LinRegPCR determines the baseline fluorescence per sample and sets a window-of-linearity (number of points between 4-6 and the best correlation coefficient) and fluorescence threshold per amplicon group. Samples with no amplification or with an efficiency 10% outside the group mean were excluded from the calculation of the mean efficiency. Statistics on the mean efficiency per amplicon are presented in the Additional file 5. The final PCR efficiency per gene is then equal to the mean PCR efficiency of all samples per amplicon.

The Pfaffl method [85] was applied to calculate the relative expression of each gene as described by Bonnet et *al.* [83]. The stability of six potential reference genes (*TBP*, *eEF1*, *SDHA*, *RPL27*, *RLP32*, *RPL19*), selected among the most stable gene expressions from placenta and endometrium literature, was tested using the GeNorm (version 3.4) algorithm [86]. The three most stable genes were selected as reference genes (*RPL19, eEF1* and *RPL32)*. Finally, the relative expression was normalized by the corresponding geometric average of these three reference genes (Additional file 6).

### Fetal phenotypes

Arterial blood samples were taken from the umbilical cord of lived fetuses. Physiological parameters (IGF1, glucose, lactate, albumin, fructose, and cortisol) were measured in arterial plasma to assess energy reserve [26]. Plasma glucose, lactate, fructose, and albumin were determined by automated enzymatic methods using a Kone multichannel analyzer. IGF-I was determined by a validated double-antibody RIA. Glycogen was assayed after extraction by homogenization with 5% trichloroacetic acid solution. Plasma cortisol was measured by direct automated immunoassay (AIA-1800, Tosoh Bioscience, San Francisco, CA).

Individual placental weights were measured for each fetus. Placental area and width were determined by picture imaging. Placental efficiency is the ratio of fetal weight (g) to placental weight (g). Other fetal biometric measures were body weight, body length, body mass index (BMI) and ponderal index (PI)).

### Statistical analysis

Statistical analyses were performed using the R 4.02 statistical software system (the Comprehensive R Archive National, http://www.cran.r-project.org).

The normalized relative expressions were transformed by a qqnorm function to conform to a normal distribution before statistical analyses. The normality of the data was checked with Shapiro and Kolmogorov–Smirnov tests. Both tests obtained a *p*-value > 0.05.

To analyze global differences between breeds and days of gestation (Table 1), the following linear models were fitted to each of the 39 genes (R lme function, lme4_1.1-28, R package) with the sow as random effect (Re) to account for repeated measures for the common maternal environment:

- Model 1) a full model including "day of gestation" factor (2 levels), "breed" factor (2 levels) and their interactions + Re,
- Model 2) an additive model including " day of gestation" factor and "breed" factor + Re,
- Model 3) a mother breed model + Re,
- Model 4) a “day of gestation” model + Re.

A F-test (ANOVA function) was performed by comparing the models and the reduced models. *P* values were corrected for test multiplicity using the Benjamini-Hochberg correction [87] available in the multtest_2.46.0 R package. The 22 genes with a significant difference (FDR< 0.05) for full or additive models were considered as differentially expressed genes (DEGs) (Table 1) and selected for further analyses.

Following the same strategy, the F-test was applied to the different subset data using appropriate mixed linear models such as:

- Model 3 for each day of gestation separately (Table 2),
- Model 4 for each breed separately (Table 2)
- Model 5) a full model including fetal genotype, sex and their interactions + Re at D90 (Table 3)
- Model 6) an additive model including " fetal genotype" factor and "sex" factor + Re at D90 (Table 3)
- Model 7) a genotype model + Re at D90 (Table 3)
- Model 8) a sex model + Re at D90 that is not presented in Table 3 because of no significativity.

A Tukey post hoc test was performed on the DEGs from models 5 and 6 to specify the significant pairwise comparisons in each breed subset.

In all experiments, results were considered significant when FDR ≤ 0.05, trend when FDR was between 0.05 and 0.1 and not significant when *FDR* ≥ 0.1.

The calculated fold change corresponds to the ratio between the mean normalized expression for each comparison.

Principal component analysis (PCA) was performed on the 22 DEGs using the packages FactoMineR_2.4 and factoextra_1.0.7. Concentration ellipses were plotted around each group mean point with an ellipse level of 0.75.

Correlation matrices were constructed from the 22 normalized relative gene expressions using the function rcorr (R package Hmisc_4.6-0) with the spearman method, clustered using ward method and plotted with the function corrplot (R package Hmisc_4.6-0). Only the correlations with a *P*-value < 0.01 are reported.

A correlation bipartite network analysis between the 22 DEG and biometric measures was performed using the mixOmics_6.14.1 package (http://www.mixOmics.org) [88, 89] according to a regression mode PLS and a specific correlation cutoff for fetal or placental measures.

### Supplementary Information

**Supplementary Material 1.**

**Supplementary Material 2.**

**Supplementary Material 3.**

**Supplementary Material 4.**

**Supplementary Material 5.**

**Supplementary Material 6.**

## Data Availability

The data supporting these results are included within the article (Additional file 4).

## References

[1] Bazer FW, Johnson GA (2014). Pig blastocyst-uterine interactions. Differentiation..

[2] Fazleabas AT, Strakova Z (2002). Endometrial function: cell specific changes in the uterine environment. Mol Cell Endocrinol.

[3] Ng SW, Norwitz GA, Pavlicev M, Tilburgs T, Simón C, Norwitz ER (2020). Endometrial Decidualization: The Primary Driver of Pregnancy Health. Int J Mol Sci.

[4] Town SC, Putman CT, Turchinsky NJ, Dixon WT, Foxcroft GR (2004). Number of conceptuses in utero affects porcine fetal muscle development. Reproduction.

[5] Riddersholm KV, Bahnsen I, Bruun TS, de Knegt LV, Amdi C (2021). Identifying Risk Factors for Low Piglet Birth Weight, High Within-Litter Variation and Occurrence of Intrauterine Growth-Restricted Piglets in Hyperprolific Sows. Animals (Basel).

[6] Foxcroft GR, Dixon WT, Novak S, Putman CT, Town SC, Vinsky MD (2006). The biological basis for prenatal programming of postnatal performance in pigs. J Anim Sci.

[7] Muns R, Nuntapaitoon M, Tummaruk P (2016). Non-infectious causes of pre-weaning mortality in piglets. Livestock Science.

[8] Leenhouwers JI, Knol EF, de Groot PN, Vos H, van der Lende T (2002). Fetal development in the pig in relation to genetic merit for piglet survival. J Anim Sci.

[9] Canario L, Père MC, Tribout T, Thomas F, David C, Gogué J, Herpin P, Bidanel JP, Le Dividich J (2007). Estimation of genetic trends from 1977 to 1998 of body composition and physiological state of Large White pigs at birth. Animal.

[10] Voillet V, SanCristobal M, Lippi Y, Martin PG, Iannuccelli N, Lascor C, Vignoles F, Billon Y, Canario L, Liaubet L (2014). Muscle transcriptomic investigation of late fetal development identifies candidate genes for piglet maturity. BMC Genomics.

[11] Lefort G, Servien R, Quesnel H, Billon Y, Canario L, Iannuccelli N, Canlet C, Paris A, Vialaneix N, Liaubet L: The maturity in fetal pigs using a multi-fluid metabolomic approach. In*.*https://www.biorxiv.org/content/10.1101/2020.03.13.990564v1; 2020. 10.1038/s41598-020-76709-8 PMC7670440 33199811

[12] Kim M, Seo H, Choi Y, Yoo I, Seo M, Lee CK, Kim H, Ka H (2015). Analysis of Stage-Specific Gene Expression Profiles in the Uterine Endometrium during Pregnancy in Pigs. PLoS One.

[13] Vélez C, Clauzure M, Williamson D, García M, Koncurat M, Barbeito C (2023). Integrins and ligands, are correlated at pig placental interface during pregnancy?. Reprod Fertil.

[14] Wang KJ, Yang KJ, Xu Q, Liu YF, Li WT, Bai Y, Wang J, Ding C, Liu XM, Tang QG (2019). Protein expression profiles in Meishan and Duroc sows during mid-gestation reveal differences affecting uterine capacity, endometrial receptivity, and the maternal-fetal Interface. Bmc Genomics.

[15] Chen F, Wang TJ, Feng CP, Lin G, Zhu YH, Wu GY, Johnson G, Wang JJ (2015). Proteome Differences in Placenta and Endometrium between Normal and Intrauterine Growth Restricted Pig Fetuses. PLoS One.

[16] Sferruzzi-Perri AN, López-Tello J, Fowden AL, Constancia M (2016). Maternal and fetal genomes interplay through phosphoinositol 3-kinase(PI3K)-p110α signaling to modify placental resource allocation. Proc Natl Acad Sci U S A.

[17] Mansouri-Attia N, Sandra O, Aubert J, Degrelle S, Everts RE, Giraud-Delville C, Heyman Y, Galio L, Hue I, Yang X (2009). Endometrium as an early sensor of in vitro embryo manipulation technologies. Proc Natl Acad Sci U S A.

[18] Petry CJ, Ong KK, Dunger DB (2007). Does the fetal genotype affect maternal physiology during pregnancy?. Trends Mol Med.

[19] Petry CJ, Koulman A, Lu L, Jenkins B, Furse S, Prentice P, Matthews L, Hughes IA, Acerini CL, Ong KK (2018). Associations between the maternal circulating lipid profile in pregnancy and fetal imprinted gene alleles: a cohort study. Reprod Biol Endocrinol.

[20] Stenhouse C, Hogg CO, Ashworth CJ (2019). Novel relationships between porcine fetal size, sex, and endometrial angiogenesis†. Biol Reprod.

[21] Stenhouse C, Hogg CO, Ashworth CJ (2019). Association of foetal size and sex with porcine foeto-maternal interface integrin expression. Reproduction.

[22] Moore GE, Oakey R (2011). The role of imprinted genes in humans. Genome Biol.

[23] O'Doherty AM, O'Shea LC, Sandra O, Lonergan P, Fair T, Forde N (2017). Imprinted and DNA methyltransferase gene expression in the endometrium during the pre- and peri-implantation period in cattle. Reprod Fertil Dev.

[24] Gao F, Ma X, Rusie A, Hemingway J, Ostmann AB, Chung D, Das SK (2012). Epigenetic changes through DNA methylation contribute to uterine stromal cell decidualization. Endocrinology.

[25] Constância M, Angiolini E, Sandovici I, Smith P, Smith R, Kelsey G, Dean W, Ferguson-Smith A, Sibley CP, Reik W (2005). Adaptation of nutrient supply to fetal demand in the mouse involves interaction between the Igf2 gene and placental transporter systems. Proc Natl Acad Sci U S A.

[26] Yao Y, Voillet V, Jegou M, SanCristobal M, Dou S, Romé V, Lippi Y, Billon Y, Père MC, Boudry G (2017). Comparing the intestinal transcriptome of Meishan and Large White piglets during late fetal development reveals genes involved in glucose and lipid metabolism and immunity as valuable clues of intestinal maturity. BMC Genomics.

[27] Vonnahme KA, Lemley CO (2012). Programming the offspring through altered uteroplacental hemodynamics: how maternal environment impacts uterine and umbilical blood flow in cattle, sheep and pigs. Reproduction Fertility and Development.

[28] Mortlock S, McKinnon B, Montgomery GW (2021). Genetic Regulation of Transcription in the Endometrium in Health and Disease. Front Reprod Health.

[29] Chen K, Chen X, He J, Ding Y, Geng Y, Liu S, Liu X, Wang Y (2015). Mouse Endometrium Temporal and Spatial Expression mRNA and MicroRNA Associated With Embryo Implantation. Reprod Sci.

[30] Fowden AL, Coan PM, Angiolini E, Burton GJ, Constancia M (2011). Imprinted genes and the epigenetic regulation of placental phenotype. Prog Biophys Mol Biol.

[31] Hanna CW (2020). Placental imprinting: Emerging mechanisms and functions. PLoS Genet.

[32] Jousse C, Bruhat A, Ferrara M, Fafournoux P (2000). Evidence for multiple signaling pathways in the regulation of gene expression by amino acids in human cell lines. J Nutr.

[33] Lomelino CL, Andring JT, McKenna R, Kilberg MS (2017). Asparagine synthetase: Function, structure, and role in disease. J Biol Chem.

[34] Strakovsky RS, Zhou D, Pan YX (2010). A low-protein diet during gestation in rats activates the placental mammalian amino acid response pathway and programs the growth capacity of offspring. J Nutr.

[35] Goodband R. D. , Tokach M. D. , Goncalves M. A. D. , Woodworth J. C. , Dritz S. S., M. DJ: Nutritional enhancement during pregnancy and its effects on reproduction in swine. In*.*, vol. 3, issue 4: Animal Frontiers 2013;68:75.

[36] Kim SW, Weaver AC, Shen YB, Zhao Y (2013). Improving efficiency of sow productivity: nutrition and health. J Anim Sci Biotechnol.

[37] Udagawa K, Miyagi Y, Hirahara F, Miyagi E, Nagashima Y, Minaguchi H, Misugi K, Yasumitsu H, Miyazaki K (1998). Specific expression of PP5/TFPI2 mRNA by syncytiotrophoblasts in human placenta as revealed by in situ hybridization. Placenta.

[38] Udagawa K, Yasumitsu H, Esaki M, Sawada H, Nagashima Y, Aoki I, Jin M, Miyagi E, Nakazawa T, Hirahara F (2002). Subcellular localization of PP5/TFPI-2 in human placenta: a possible role of PP5/TFPI-2 as an anti-coagulant on the surface of syncytiotrophoblasts. Placenta.

[39] Chu AJ (2011). Tissue factor, blood coagulation, and beyond: an overview. Int J Inflam.

[40] Klaitman V, Beer-Wiesel R, Rafaeli T, Mazor M, Erez O: The Role of the Coagulation System in Preterm Parturition, Preterm Birth. In*.* Edited by Offer E: IntechOpen; January 23rd 2013.

[41] Lockwood CJ, Krikun G, Schatz F (1999). The decidua regulates hemostasis in human endometrium. Semin Reprod Endocrinol.

[42] Mayer W, Hemberger M, Frank HG, Grümmer R, Winterhager E, Kaufmann P, Fundele R (2000). Expression of the imprinted genes MEST/Mest in human and murine placenta suggests a role in angiogenesis. Dev Dyn.

[43] Peng W, Chen Y, Luo X, Shan N, Lan X, Olson D, Zhang H, Ding YB, Qi HB (2016). DNA methylation-associated repression of MEST/PEG1 expression contributes to the invasion of extravillous trophoblast cells. Placenta.

[44] Hansson SR, Bottalico B, Noskova V, Casslén B (2009). Monoamine transporters in human endometrium and decidua. Hum Reprod Update.

[45] Karahoda R, Horackova H, Kastner P, Matthios A, Cerveny L, Kucera R, Kacerovsky M, Duintjer Tebbens J, Bonnin A, Abad C (2020). Serotonin homeostasis in the materno-foetal interface at term: Role of transporters (SERT/SLC6A4 and OCT3/SLC22A3) and monoamine oxidase A (MAO-A) in uptake and degradation of serotonin by human and rat term placenta. Acta Physiol (Oxf).

[46] Biensen NJ, Wilson ME, Ford SP (1999). The impacts of uterine environment and fetal genotype on conceptus size and placental vascularity during late gestation in pigs. J Anim Sci.

[47] Yang K, Wang J, Wang K, Luo Y, Tang Q, Liu X, Fang M (2020). Integrated Analysis of miRNA-mRNA Network Reveals Different Regulatory Patterns in the Endometrium of Meishan and Duroc Sows during Mid-Late Gestation. Animals (Basel).

[48] Luhmann UF, Meunier D, Shi W, Lüttges A, Pfarrer C, Fundele R, Berger W (2005). Fetal loss in homozygous mutant Norrie disease mice: a new role of Norrin in reproduction. Genesis.

[49] Jouannet S, Saint-Pol J, Fernandez L, Nguyen V, Charrin S, Boucheix C, Brou C, Milhiet PE, Rubinstein E (2016). TspanC8 tetraspanins differentially regulate the cleavage of ADAM10 substrates, Notch activation and ADAM10 membrane compartmentalization. Cell Mol Life Sci.

[50] Koh YQ, Peiris HN, Vaswani K, Reed S, Rice GE, Salomon C, Mitchell MD (2016). Characterization of exosomal release in bovine endometrial intercaruncular stromal cells. Reprod Biol Endocrinol.

[51] Zhao S, Gu Y, Fan R, Groome LJ, Cooper D, Wang Y (2010). Proteases and sFlt-1 release in the human placenta. Placenta.

[52] Maynard SE, Venkatesha S, Thadhani R, Karumanchi SA (2005). Soluble Fms-like tyrosine kinase 1 and endothelial dysfunction in the pathogenesis of preeclampsia. Pediatr Res.

[53] Lala PK, Nandi P (2016). Mechanisms of trophoblast migration, endometrial angiogenesis in preeclampsia: The role of decorin. Cell Adh Migr.

[54] Nandi P, Siddiqui MF, Lala PK (2016). Restraint of Trophoblast Invasion of the Uterus by Decorin: Role in Pre-eclampsia. Am J Reprod Immunol.

[55] Menchini RJ, Chaudhry FA (2019). Multifaceted regulation of the system A transporter Slc38a2 suggests nanoscale regulation of amino acid metabolism and cellular signaling. Neuropharmacology.

[56] Gibson C, de Ruijter-Villani M, Rietveld J, Stout TAE (2018). Amino acid transporter expression in the endometrium and conceptus membranes during early equine pregnancy. Reprod Fertil Dev.

[57] Cramer S, Beveridge M, Kilberg M, Novak D (2002). Physiological importance of system A-mediated amino acid transport to rat fetal development. Am J Physiol Cell Physiol.

[58] Père MC, Etienne M, Dourmad JY (2000). Adaptations of glucose metabolism in multiparous sows: effects of pregnancy and feeding level. J Anim Sci.

[59] Père MC, Etienne M (2019). Influence of litter size on insulin sensitivity in multiparous sows. J Anim Sci.

[60] Pantaleon M, Kaye PL (1998). Glucose transporters in preimplantation development. Rev Reprod.

[61] Dean M (2019). Glycogen in the uterus and fallopian tubes is an important source of glucose during early pregnancy†. Biol Reprod.

[62] Charalambous M, Smith FM, Bennett WR, Crew TE, Mackenzie F, Ward A (2003). Disruption of the imprinted Grb10 gene leads to disproportionate overgrowth by an Igf2-independent mechanism. Proc Natl Acad Sci U S A.

[63] Charalambous M, Cowley M, Geoghegan F, Smith FM, Radford EJ, Marlow BP, Graham CF, Hurst LD, Ward A (2010). Maternally-inherited Grb10 reduces placental size and efficiency. Dev Biol.

[64] Canario L, Billon Y, Caritez JC, Bidanel JP, Laloe D (2009). Comparison of sow farrowing characteristics between a Chinese breed and three French breeds. Livestock Science.

[65] Gu T, Su X, Zhou Q, Li X, Yu M, Ding Y, Zhao S, Li C (2012). Molecular characterization of the Neuronatin gene in the porcine placenta. PLoS One.

[66] Xing P, Hong L, Yan G, Tan B, Qiao J, Wang S, Li Z, Yang J, Zheng E, Cai G (2022). Neuronatin gene expression levels affect foetal growth and development by regulating glucose transport in porcine placenta. Gene.

[67] Miller SG, Hafen PS, Law AS, Springer CB, Logsdon DL, O'Connell TM, Witczak CA, Brault JJ (2021). AMP deamination is sufficient to replicate an atrophy-like metabolic phenotype in skeletal muscle. Metabolism.

[68] Sahlin K, Broberg S (1990). Adenine nucleotide depletion in human muscle during exercise: causality and significance of AMP deamination. Int J Sports Med.

[69] Roszkowska A, Klimek J, Kaletha K (2008). Expression patterns of AMP-deaminase and cytosolic 5'-nucleotidase genes in human term placenta. Mol Cell Biochem.

[70] Steinhauser CB, Landers M, Myatt L, Burghardt RC, Vallet JL, Bazer FW, Johnson GA (2016). Fructose Synthesis and Transport at the Uterine-Placental Interface of Pigs: Cell-Specific Localization of SLC2A5, SLC2A8, and Components of the Polyol Pathway. Biol Reprod.

[71] Bowman CE, Arany Z, Wolfgang MJ (2021). Regulation of maternal-fetal metabolic communication. Cell Mol Life Sci.

[72] Wilson ME, Biensen NJ, Youngs CR, Ford SP (1998). Development of Meishan and Yorkshire littermate conceptuses in either a Meishan or Yorkshire uterine environment to day 90 of gestation and to term. Biol Reprod.

[73] Al-Qaraghouli M, Fang YMV (2017). Effect of Fetal Sex on Maternal and Obstetric Outcomes. Front Pediatr.

[74] Dearden L, Bouret SG, Ozanne SE (2018). Sex and gender differences in developmental programming of metabolism. Mol Metab.

[75] Christians JK (2021). The Placenta's Role in Sexually Dimorphic Fetal Growth Strategies. Reprod Sci.

[76] Buckberry S, Bianco-Miotto T, Bent SJ, Dekker GA, Roberts CT (2014). Integrative transcriptome meta-analysis reveals widespread sex-biased gene expression at the human fetal-maternal interface. Mol Hum Reprod.

[77] Stenhouse C, Hogg CO, Ashworth CJ: Associations between fetal size, sex and both proliferation and apoptosis at the porcine feto-maternal interface. In*.*, vol. 70: Placenta; 2018: 15-24. 10.1016/j.placenta.2018.08.006 PMC6215148 30316322

[78] Chao W, D'Amore PA (2008). IGF2: epigenetic regulation and role in development and disease. Cytokine Growth Factor Rev.

[79] Angiolini E, Coan PM, Sandovici I, Iwajomo OH, Peck G, Burton GJ, Sibley CP, Reik W, Fowden AL, Constância M (2011). Developmental adaptations to increased fetal nutrient demand in mouse genetic models of Igf2-mediated overgrowth. FASEB J.

[80] Stenhouse C, Bazer FW, Ashworth CJ (2023). Sexual dimorphism in placental development and function: Comparative physiology with an emphasis on the pig. Mol Reprod Dev.

[81] Mansouri-Attia N, Aubert J, Reinaud P, Giraud-Delville C, Taghouti G, Galio L, Everts RE, Degrelle S, Richard C, Hue I (2009). Gene expression profiles of bovine caruncular and intercaruncular endometrium at implantation. Physiol Genomics.

[82] Voillet V, San Cristobal M, Père MC, Billon Y, Canario L, Liaubet L, Lefaucheur L (2018). Integrated Analysis of Proteomic and Transcriptomic Data Highlights Late Fetal Muscle Maturation Process. Mol Cell Proteomics.

[83] Bonnet A, Cabau C, Bouchez O, Sarry J, Marsaud N, Foissac S, Woloszyn F, Mulsant P, Mandon-Pepin B (2013). An overview of gene expression dynamics during early ovarian folliculogenesis: specificity of follicular compartments and bi-directional dialog. BMC Genomics.

[84] Ruijter JM, Ramakers C, Hoogaars WM, Karlen Y, Bakker O, van den Hoff MJ, Moorman AF (2009). Amplification efficiency: linking baseline and bias in the analysis of quantitative PCR data. Nucleic Acids Res.

[85] Pfaffl MW (2001). A new mathematical model for relative quantification in real-time RT-PCR. Nucleic Acids Res.

[86] Vandesompele J, De Preter K, Pattyn F, Poppe B, Van Roy N, De Paepe A, Speleman F (2002). Accurate normalization of real-time quantitative RT-PCR data by geometric averaging of multiple internal control genes. Genome Biol.

[87] Benjamini V, Hochberg V (1995). Controlling the false discovery rate : a practical and powerful approach to multiple testing. J R Statist Soc B.

[88] Gonzalez I, Le Cao KA, Davis MJ, Dejean S (2012). Visualising associations between paired 'omics' data sets. Biodata Mining.

[89] Rohart F, Gautier B, Singh A, Le Cao KA (2017). mixOmics: An R package for 'omics feature selection and multiple data integration. Plos Computational Biology.

