# Supplementary material for: Sex and fetal genome influence gene expression in pig endometrium at the end of gestation
Source: BMC Genomics. 2024 Mar 21;25:303. doi: 10.1186/s12864-024-10144-1 (PMC10958934; doi:10.1186/s12864-024-10144-1)
Supplement: Supplementary file 3 — Supplementary Material 3. [file 12864_2024_10144_MOESM3_ESM.pdf]

A : NDP

B : IGF2R

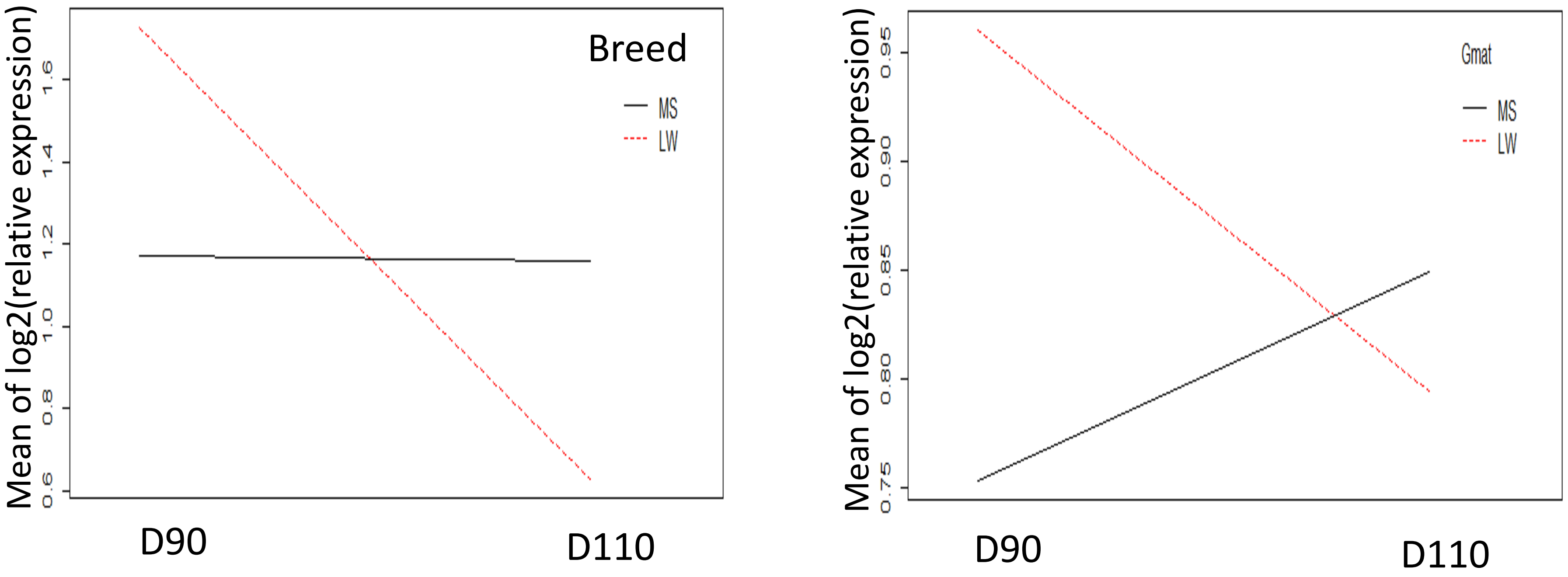

Additional file 3 – Relative expression of NDP and IGF2R genes

Expression of *NDP* and *IGF2R* genes varied by day of gestation and breed.

The Y-axis corresponds to the endometrium normalized relative expression.
The X-axis corresponds to the two days of gestation.
